# Neutrophil-to-lymphocyte ratio and platelet-to-lymphocyte ratio as potential diagnostic markers for rebleeding in patients with esophagogastric variceal bleeding

**DOI:** 10.1515/biol-2022-0852

**Published:** 2024-08-23

**Authors:** Lei Chen, Cong Tong, Xiangan Zhao, Chunfang Xu

**Affiliations:** Department of Gastroenterology, The First Affiliated Hospital of Soochow University, Suzhou, Jiangsu 215006, P.R. China; Department of Gastroenterology, Northern Jiangsu People’s Hospital Affiliated to Yangzhou University, Yangzhou, Jiangsu 225001, P.R. China

**Keywords:** esophagogastric variceal bleeding, rebleeding, NLR, PLR, diagnosis

## Abstract

The aim of the present study is to explore the potential prediction value of neutrophil-to-lymphocyte ratio (NLR) or peripheral blood platelet-to-lymphocyte ratio (PLR) for rebleeding in patients with esophagogastric variceal bleeding (EVB). We have enrolled 80 rebleeding patients with EVB and 113 EVB patients without rebleeding in the present study. The lymphocyte, platelet counts, the PLR, and the NLR of the candidates were calculated, and receiver-operating characteristic curve was drawn to examine whether NLR or PLR is a sensitive biomarker for distinguishing rebleeding patients from the EVB patients. We observed that NLR and PLR were all significantly increased in rebleeding patients with EVB compared with the non-rebleeding patients (*p* < 0.01); moreover, the area under the curve of NLR and PLR was 0.7037 (95% confidence interval [CI], 0.6281–0.7792) and 0.7468 (95% CI, 0.6793–0.8144), respectively, suggesting that NLR or PLR is a sensitive biomarker for distinguishing non-rebleeding patients from the rebleeding patients. We reported that NLR and PLR were significantly increased in the peripheral blood of patient with esophagogastric variceal rebleeding, suggesting that NLR and PLR may be potential early diagnostic and prognostic markers for the rebleeding among patients with EVB.

## 1 Introduction

Esophagogastric variceal bleeding (EVB) is one of the most common and life-threatening complications of portal hypertension in cirrhosis [1]. Although the case fatality rate of EVB has decreased significantly compared to more than 40% in the last century, the case fatality rate is still 15–20% within 6 weeks [2]. In particular, the case fatality rate in patients with heavy bleeding is as high as more than 40% [3]. Once variceal bleeding occurs, the rebleeding rate can reach 60–80% within 1 year, which seriously affects the quality of life of patients with cirrhosis and increases medical expenses [4].

EVB is characterized by a rapid onset, rapid progression, dangerous disease, and high mortality, therefore, if high-risk patients can be screened as early as possible and given appropriate standardized treatment, the occurrence of adverse events can be reduced to a certain extent [5]. In recent years, many literature and related expert consensus and guidelines recommend drug combination with endoscopic variceal ligation (EVL) treatment as a first-line regimen, which has the advantages of high success rate of hemostasis and small side effects, but the recurrence rate remains high [6]. In general, it is recommended to perform the second ligation 4 weeks after the first band, and then the third banding every 2 months, and then the interval is 3 months, so long-term follow-up and timely follow-up treatment are important to prevent rebleeding [7]. However, in actual clinical work, there are limited factors such as poor patient compliance and heavy economic burden. Physicians are passive in re-intervention after acute EVL treatment, and although there is theoretical support, there is a lack of simple and cost-effective indicators that effectively predict the risk of rebleeding. In particular, such patients are admitted to the hospital in an acute and unstable condition, which limits the improvement of invasive, complex and moving examination items for patients [8].

In recent years, studies on the serological parameters for the early diagnosis of different disease have become an area of focus. Some previous studies demonstrated that neutrophil-to-lymphocyte ratio (NLR) or platelet-to-lymphocyte ratio (PLR) was significantly increased in patients with different types of diseases [9,10], for example, cancer, cardiovascular disease, and autoimmune diseases, suggesting that either NLR [11,12,13,14] or PLR [15,16,17] may serve as novel biomarkers for the early diagnosis of those diseases. However, there is currently no research on the NLR and PLR in the diagnosis and treatment of EVB. This study designed to explore the NLR and PLR in the diagnosis of EVB.

At present, multiple literature reports on the influence factors of EVB at home and abroad, but the influencing factors of EVB rebleeding have rarely been reported [18]. Hence, we conducted this study to explore the potential diagnostic markers for rebleeding in patients with EVB, with the aim to provide appropriate prevention and treatment measures for the clinical treatment of rebleeding after treatment of EVB. This study may provide novel evidence for the application of PLR and NLR as potential biomarkers for the early diagnosis of esophagogastric variceal rebleeding.

## 2 Methods

### 2.1 Subjects

A retrospective analysis was performed on the clinical data of patients with esophageal variceal bleeding with cirrhosis who underwent endoscopic polylauryl alcohol combined with therapy in our hospital from January 2014 to December 2016, and the follow-up time was 4 years after treatment. Inclusion criteria were as follows: (1) patients with clinical manifestations of upper gastrointestinal bleeding, such as hematemesis, melena, and peripheral volume depletion; (2) the presence of esophageal varices with or without fundus varices was confirmed by endoscopy; (3) treatment methods refer to the requirements of 2016 “Guidelines for the diagnosis and treatment of esophageal and gastric variceal bleeding in cirrhotic portal hypertension” [19]; and (4) patients have complete medical records and clinical treatment data. Exclusion criteria were as follows: (1) patients complicated with malignant tumors, blood diseases, primary kidney disease, and severe cardiovascular diseases; (2) patients with simple gastric varices; and (3) patients with incomplete clinical data or lost follow-up.


**Informed consent:** Informed consent has been obtained from all individuals included in this study.
**Ethical approval:** The research related to human use has been complied with all the relevant national regulations, institutional policies and in accordance with the tenets of the Helsinki Declaration, and has been approved by the Ethics Committee of the First Affiliated Hospital of Soochow University Hospital.

### 2.2 Criteria for rebleeding

After the first endoscopic treatment, patients with esophageal and gastric varices experienced active bleeding again, and the cause of the bleeding was confirmed to be rupture and bleeding of esophageal and gastric varices by gastroscopy under the stable hemodynamic state within 24 h.

### 2.3 Clinical laboratory analysis

The peripheral blood of the patients and the healthy volunteers were collected and examined at the Clinical Laboratory Department of the First Affiliated Hospital of Soochow University Hospital for the serological analysis. The samples of the patients were collected when they first came to the outpatient department of the First Affiliated Hospital of Soochow University Hospital without any treatment. The lymphocyte counts and platelet counts were recorded, and the NLR and PLR were calculated.

### 2.4 Statistical analysis

SPSS25.0 statistical software was used for statistical analysis, Chi-square test or Fisher exact probability method was used for comparison between counting data groups, and Wilcoxon rank sum test was used for rank classification data. The indicators that are meaningful for univariate analysis are included in the model, and binary logistic regression analysis is carried out with rebleeding as the dependent variable, and the stepwise entry method is adopted to obtain independent influence factors.

## 3 Results

### 3.1 Clinical characteristics of the patients

According to the inclusion and exclusion criteria of this study, a total of 193 patients (including 113 non-rebleeding and 80 rebleeding patients) with liver cirrhosis were included through the electronic information management system of patients admitted to our hospital. They all received secondary prevention of variceal rebleeding and transient elastic imaging measurement of liver. For the non-rebleeding group, the male/female ratio is 67/46, and the average age of the patients was 61.39 ± 12.97. In terms of the etiology of liver cirrhosis, there are 15 cases of hepatitis B; patients with cirrhosis accounted for 106. For the rebleeding group, the male/female ratio is 56/24, and the average age of the patients was 64.65 ± 12.30. In terms of the etiology of liver cirrhosis, there are 7 cases of hepatitis B; patients with cirrhosis accounted for 68.

#### 3.1.1 Elevated PLR count and NLR in EVB patients with rebleeding

**Figure 1:**
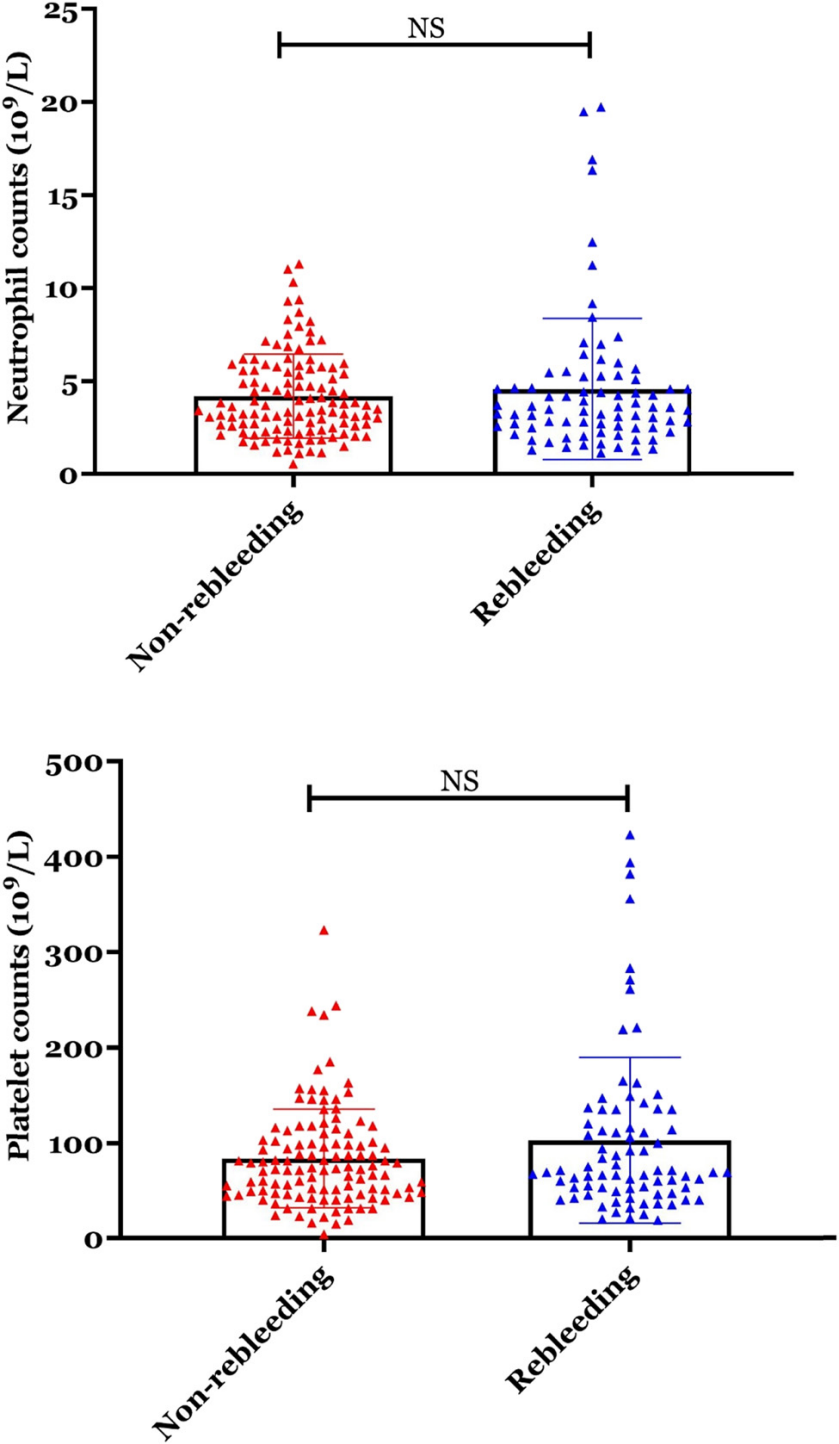
Neutrophil counts and platelet count showed no significant differences between the two groups.

**Figure 2:**
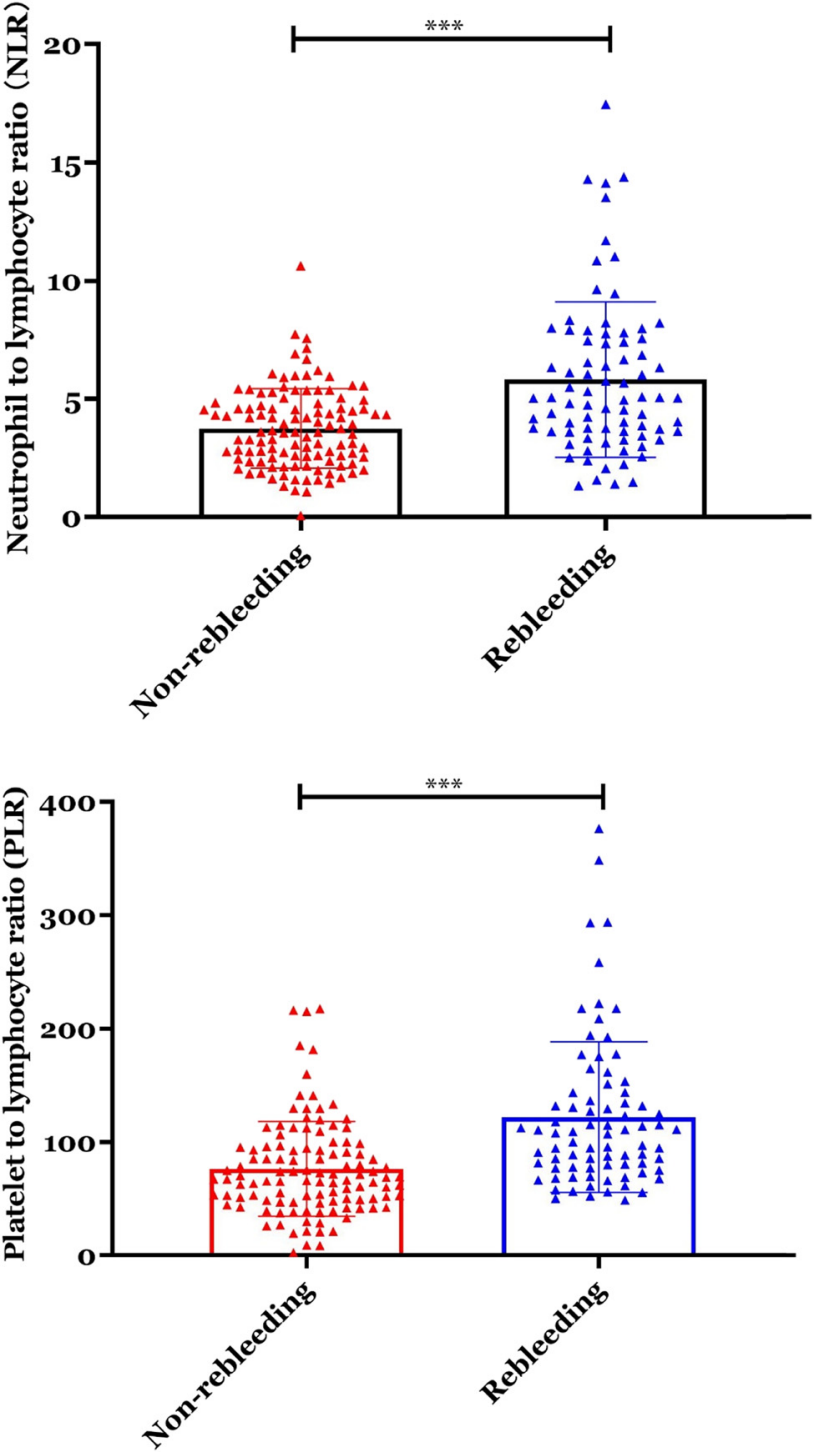
NLR and PLR were all significantly increased in the rebleeding group compared with the non-rebleeding group. ***p < 0.01.

Next, we have analyzed the serological data of participators between the EVB patients with rebleeding and non-rebleeding groups. The neutrophil counts and platelet count showed no significant differences between the two groups (Figure 1, *p* > 0.05). On the other hand, the NLR and PLR were all significantly increased in the rebleeding group compared with the non-rebleeding group (Figure 2, *p* < 0.01).

#### 3.1.2 NLR and PLR as early diagnostic markers in EVB patients with rebleeding

**Figure 3:**
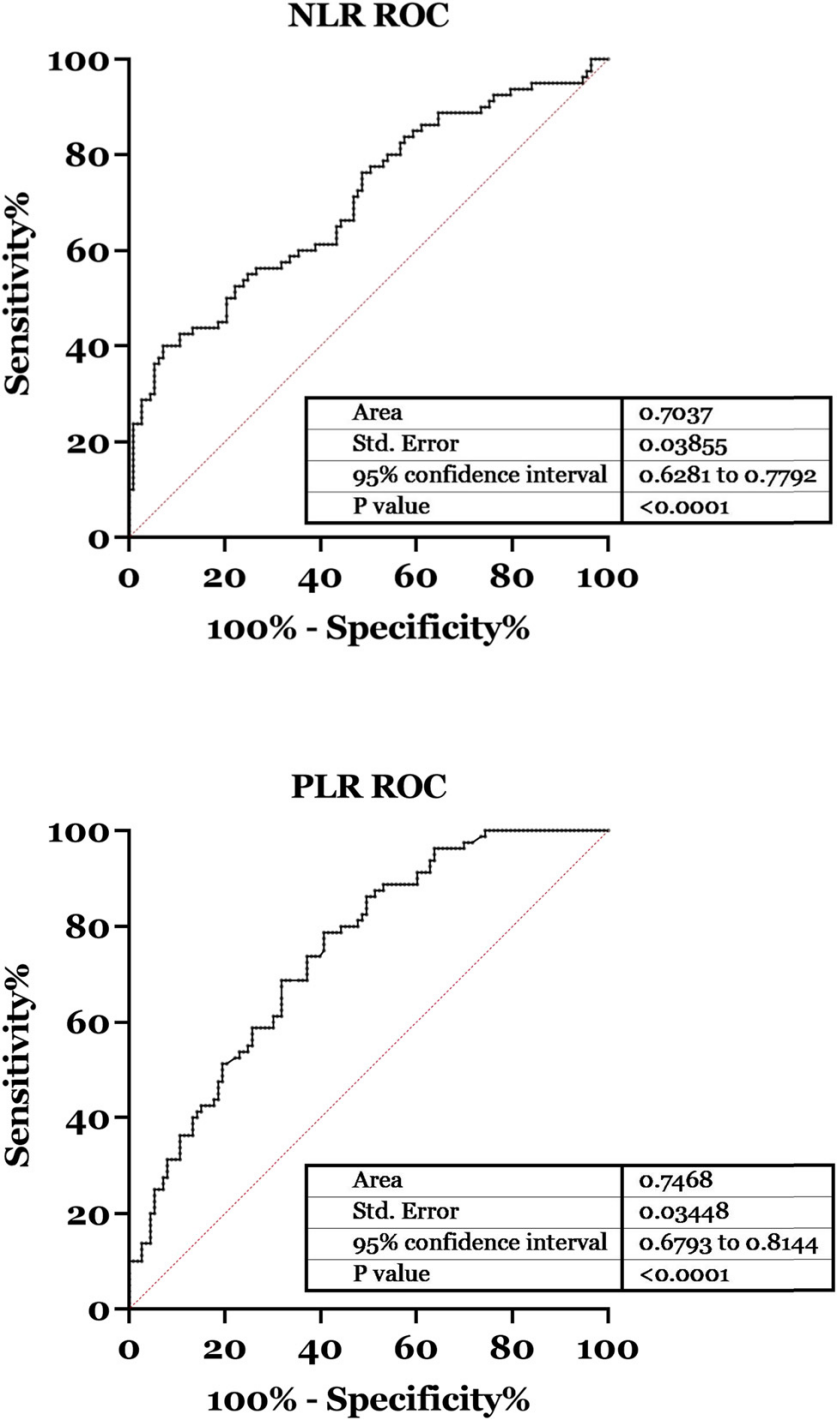
ROC curves for NLR and PLR.

Finally, the receiver-operating characteristic (ROC) curves have been drawn to show the sensitivity and specificity of NLR and PLR to distinguish rebleeding patients from non-rebleeding patients. As shown in Figure 3, the area under the curve (AUC) of PLR was 0.7468 (95% confidence interval [CI], 0.6793 to 0.8144), and the AUC of NLR was 0.7037 (95% CI, 0.6281 to 0.7792), respectively.

## 4 Discussion

In the present study, the roles of NLR and PLR as potential early diagnostic and prognostic markers for EVB patients with rebleeding have been discussed. We proved that both NLR and PLR were significantly upregulated in EVB patients with rebleeding, and NLR and PLR are sensitive diagnostic and prognostic markers for rebleeding. Our results have proposed the potential diagnostic value of NLR and PLR for the early diagnosis and prognostic marker of EVB patients with rebleeding.

At present, transient elastic imaging technology is used to measure liver stiffness (LSM) in clinics, which has excellent performance in predicting portal hypertension. On one hand, LSM can distinguish patients with or without clinically significant portal hypertension, and the area under the ROC curve ranges from 0.82 to 0.94 [20]; on the other hand, the screening of esophageal varices requires gastroduodenoscopy at the time of diagnosis of cirrhosis, which is not the optimal solution from the perspective of economic benefits and patient acceptability. However, almost all studies focused on the predictive value of LSM for the first esophageal variceal bleeding in patients with liver cirrhosis, and there was a lack of non-invasive indicators to predict the rebleeding events in patients with liver cirrhosis undergoing secondary prevention.

Increasing evidence indicated that calculating the ratio of different immune cells, i.e., neutrophils/lymphocyte ratio, platelet/lymphocyte in the peripheral blood is a reproducible method for the early diagnosis of different diseases with low cost. PLR has been discussed in many previous studies as a potential biomarker for the diagnosis and prognosis of different diseases [21,22,23,24,25]. In the present study, as previously discussed we observed that the PLR was significantly increased in the rebleeding group compared with the non-rebleeding group (*p* < 0.01); moreover, results of ROC analysis indicated that the AUC of PLR was 0.7468. These results suggested that PLR is a sensitive biomarker to distinguish rebleeding patients from the non-rebleeding patients; moreover, ROC analysis suggested that the AUC of NLR was 0.7037. These results suggested that NLR is also a sensitive biomarker to distinguish rebleeding patients from the non-rebleeding patients.

Our studies have limitations. First, the results should be verified with lager sample size in future studies; second, this study was based on Chinese Han population, so whether NLR or PLR has diagnostic value for rebleeding after EVB in other races still need to be validated.

## 5 Conclusions

In conclusion, we proved for the first time that NLR and PLR were all significantly increased in EVB patients with rebleeding, and our results have provided novel evidence that NLR and PLR were sensitive biomarkers for the early diagnosis of rebleeding among patients with EVB.
